# Perceptions of Diabetes, Barriers to Disease Management, and Service Needs: A Focus Group Study of Working Adults with Diabetes in Hawaii

**Published:** 2011-02-15

**Authors:** Landry L. Fukunaga, Denise L. Uehara, Tammy Tom

**Affiliations:** Evaluation Specialist, Center on Disability Studies, University of Hawaii at Manoa; University of Hawaii at Manoa, Honolulu, Hawaii; University of Hawaii at Manoa, Honolulu, Hawaii

## Abstract

**Introduction:**

Research about the support needs for and barriers to successful disease management of working adults with diabetes is limited. Our objective was to gain an in-depth understanding of how working adults in Hawaii perceive diabetes, barriers to disease management, and the services needed to keep people healthy and working.

**Methods:**

From November 2008 through March 2009, we conducted focus group interviews with 74 employed adults with diabetes enrolled in the Hawaii Demonstration to Maintain Independence and Employment project. Responses to questions were analyzed within and across groups to identify recurring themes. A third layer of analysis examined themes across responses to all questions, specifically, how barriers related to identified service needs.

**Results:**

Employed participants with diabetes experienced pervasive effects on their lives as a result of the disease, although they interpreted these effects positively or negatively. Barriers to disease management, such as additional health issues, social prejudice, and lack of social support, indicated a need to educate the general public about the disease. Participants identified needing social support from other people with diabetes, psychological support to address the emotional side of diabetes, and coordinated teams of specialists to address medication side effects and other health-related barriers to disease management. Many participants discussed the challenge of integrating diabetes management with work and family responsibilities and the need for monetary support.

**Conclusion:**

This study provides insight into how employed adults perceived their disease and what they perceived as challenges to successfully managing diabetes. The findings provide future directions for community and workplace diabetes initiatives.

## Introduction

Diabetes is a chronic health condition that affects millions of Americans and increases risk for developing disease-related complications such as blindness, cardiovascular disease, renal failure, stroke, neuropathy, and amputation (1). On the basis of 2000-2002 data, an estimated 100,000 people in Hawaii had diabetes, and approximately 25% of these cases were undiagnosed (2). As of 2005, diabetes was the seventh leading cause of death in the state (3), and it is more prevalent among Native Hawaiians, Filipinos, and Japanese residents (4).

The health and employment costs of diabetes are considerable. In 2007, the estimated national cost of diabetes exceeded $174 billion. This estimate included $116 billion in diabetes-related medical costs and $58 billion in reduced productivity due to increased work absenteeism, reduced work and daily productivity, unemployment from disease-related disability, and early death (5). The increasing prevalence of diabetes means that finding practical approaches to keep people healthy and employed is imperative.

Several studies examining the diverse population of Hawaii identified contextually relevant factors necessary for diabetes management (6-8) and the prevention of chronic disease (9). However, research concerning programmatic needs of employed people with diabetes is limited. One qualitative study examined employee perceptions of education needs, but generalizability of these results were limited by the study's small sample (10).

The Hawaii Demonstration to Maintain Independence and Employment (HI-DMIE) was a federally funded, community-based randomized study that investigated whether medical assistance and other supports can forestall or prevent the loss of employment and independence due to diabetes complications. We present results from a cross-sectional secondary study to examine how working adults in Hawaii perceive diabetes, services, and barriers to disease management. Our results provide insight for future diabetes initiatives.

## Methods

### Recruitment and sample

The HI-DMIE study recruited volunteer participants through word of mouth, newspaper ads, placards in the public transportation system, and pamphlets in doctors' offices, pharmacies, human resource departments, and diabetes-related public events. Between April and September 2008, the HI-DMIE study used a 2:1 ratio stratified by diabetes type (type 1, type 2 or prediabetes) and randomly assigned 190 eligible participants into treatment (n = 128) and control (n = 62) groups. The 2:1 ratio allowed more participants the opportunity to be assigned to the treatment group. Eligibility for HI-DMIE included working a minimum of 40 hours per month, earning minimum wage or more (federal rate = $5.15/hr), being aged 18 to 62 years, living on the island of Oahu, and having a diagnosis of diabetes or a hemoglobin A1c (HbA1c) of 6.5% or more. Baseline demographic data verified that the 2 groups were similar in terms of age, sex, diabetes type, race/ethnicity, educational attainment, household income, daily functioning, and work productivity (11).

Through letters, telephone calls, and e-mail, we invited all participants to attend focus groups approximately 6 months after they enrolled in the study. Participants enrolled at different times, so we determined focus group composition by the month participants were enrolled in the study and group assignment (treatment or control). Recruitment efforts yielded a convenience sample of 74 of 186 (40%) participants who were enrolled in the study after 6 months. The sample contained 47 (64%) participants from the treatment group and 27 (36%) participants from the control group.

Focus group size ranged from 1 to 7 people; average group size was 4 participants. One focus group had only 1 person when 2 scheduled participants did not show up. All focus group participants consented to being audio recorded, and their responses were kept confidential. The institutional review board of the University of Hawaii approved the focus group study, and each person received $20 for participating.

### Data collection

HI-DMIE evaluators facilitated 18 focus groups from November 2008 through March 2009. We conducted focus groups on evenings and weekends in community settings (eg, coffee shops, restaurants, library meeting rooms) that were conducive to small-group discussions. Facilitators chose settings that allowed for uninterrupted conversation and discretion.

Two researchers attended each focus group. In keeping with a semistructured focus group format, a facilitator led each group through a set of predetermined questions with accompanying prompts that allowed for additional probes as needed. The facilitator summarized reactions to each question, and participants had an opportunity to respond to one another or add to their answers. Both researchers took notes, capturing conversational content; one researcher specifically noted interpersonal interactions, nonverbal reactions, and group dynamics. After each group, the researchers met to debrief and discuss group dynamics.

### Data analysis

**Box.** Questions From a Focus Group Study of Working Adults With Diabetes, Hawaii, 2008

Describe 1 image that comes to mind when you think about diabetes.
How does diabetes or prediabetes affect your life?
Have you ever experienced any barriers to managing your diabetes?
Are there services you need to support your diabetes management that you currently do not have?

Research assistants transcribed audio recordings verbatim and removed identifying personal information for 17 of the 18 focus groups. One audio recording was lost; however, notes taken by the facilitator and note taker provided sufficient detail to include this group's responses in the analysis. We read each transcript, independently identified recurring themes, and met to reach consensus on emergent themes. One researcher coded responses to each question (Box) by using notes pertaining to group dynamics to identify themes that occurred most frequently and compared this information within and across study groups. A third layer of analysis was used to examine themes across responses to all questions, specifically, how barriers related to service needs.

## Results

Most of the sample was older than 35 years, female, and Native Hawaiian or other Pacific Islander, and most respondents had type 2 diabetes (Table 1). There was no difference between people who participated in focus groups and those who did not in terms of age, HbA1c level, or type of diabetes (Table 2). Average self-reported duration of diabetes for focus group participants was 8.2 years (SD = 8.08, N = 70).

Treatment and control participants responded similarly within and across study groups, and some themes were discussed more extensively than others. Given the uniformity of responses, this article presents results from the third layer of analysis, which related perceptions about diabetes and barriers to management with identified service needs across all questions.

### Pervasive effects of diabetes

Many participants felt that diabetes affected all aspects of their lives because the disease is a constant, lifelong challenge. Several comments related to its negative effects, such as the inconvenience of having to plan for meals, test blood glucose, and manage fluctuating blood glucose levels, all of which restrict personal freedom. Conversely, other participants shared the positive effects of having the disease. Some participants stated that having diabetes forced them to prioritize health needs and make positive lifestyle changes. Resiliency factors included having a positive outlook, being proactive or self-motivated, and seeing diabetes management as a personal responsibility.

As 1 participant declared,

You know, I think by my choice I'm gonna choose to have it be positive. It has to be positive; it's what I'm doing for myself now. I have no choice, so it's a good thing. . . . I guess it's when you say it's tough love, you know, for myself.

### Diabetes complications and education needs

When asked to think about diabetes, participants most frequently mentioned physical complications leading to blindness and amputation. Many shared stories of family members who suffered or died from disease-related complications. Although participants frequently discussed being afraid of losing their independence and functioning in the future, few mentioned taking active steps to prevent unwanted complications.

Participants identified more education on how to prevent diabetes-related complications as a service need, specifically for family members and the public. As 1 participant stated,

I think there needs to be a different approach, as far as educating people about diabetes, because I look at . . . all of my aunties and uncles . . . they all are diabetic, down to my own brother [who] was in a diabetic coma and nobody said anything about [it], do something about it prior.

### Lack of understanding and social support needs

Participants discussed the social effects of diabetes such as feeling the need to conceal their diagnosis, dealing with judgmental reactions from others, and experiencing negative effects on social relationships. Participants experienced disease-related social stigma that resulted from having to use needles, use sick leave, and impose dietary limitations on themselves. As 1 participant shared, "My coworkers thought I was faking it. I was put in the hospital and . . . all I heard was negative comments like, 'Well, it's just diabetes, you know, how much can it affect her?'" Additional barriers to diabetes management included a lack of understanding and support from family members and coworkers, which typically related to social support for healthful eating habits.

Service needs associated with this theme encompassed educational supports for family members and the public. Some participants felt that increasing the public's understanding of the disease would alleviate social stigma and strengthen awareness. In other situations, education for the whole family related to support for making positive life changes. As 1 participant shared,

One thing I would like to see is meeting with my family. So that they understand what I need to do . . . you know, be more supportive. [Be]cause I mean, for those of us who've had it so long, your family kind of just thinks, 'They got it. Easy.' But when you make a decision to try and change and improve your lifestyle and manage the disease, I think other people need to come in and speak to those who are immediate members of their family.

### Emotional effects, psychological barriers, and social-emotional needs

A common theme across focus groups related to negative emotional effects such as fear, denial, depression, stress, anger, and irritability. Of particular consequence was the influence of diabetes on participants' emotional states, which in turn affected blood glucose levels. Both groups also discussed psychological barriers to diabetes management such as denial, depression, and "burnout." One participant disclosed, "I was in denial . . . and then the stresses from it, the stresses meaning the depression from realizing that, hey, I had diabetes. Once I acknowledged it, then I could do what I needed to do."

Emotional and psychological supports were frequently discussed and reiterated as a service need. As 1 participant stated,

The only support that I really need is basically . . . mental support, I guess, because I do suffer from depression a lot. . . . I had thoughts of committing suicide, too, because of diabetes and because of seeing family members die from diabetes, seeing it slowly, you know, watch[ing] your legs decay, and all of a sudden they just pull the plug. . . . It's really scary, you know.

Participants also emphasized a need to communicate with other people with diabetes about emotional barriers and ways to increase willpower and motivation. In response to this, participants mentioned needing social and motivational supports such as frequent support groups or a diabetes buddy. The following focus group interaction illustrates this suggestion:

P1: I find that the support I get from other people who have diabetes is the most valuable thing to me. So, how about having, like, diabetes buddies, you know, just a one-to-one kind of relationship where 2 can go to the gym together. . . . I more than likely would go to the gym more often. And I would watch my diet, too.

P2: It's just motivation, and like you said [to P1], buddy up and compar[e] notes with other people who have diabetes. You know, that would help, too.

### Health-related barriers to diabetes management and a need for coordinated services

Participants identified additional health issues as being a barrier to their diabetes management. Most comments related to physical limitations that stemmed from other illnesses or injuries that prevented regular exercise. Additional comments pertained to medication side effects, participant comorbidities, and diabetes complications.

Identified service needs that address these barriers included coordinated diabetes programs that incorporate exercise classes tailored for people with diabetes and who have varying physical abilities. Participants also frequently discussed a need for collaborative approaches to health care. In 1 focus group, participants shared the following:

P1: All the doctors that we have are experts in 1 slice of our body. So, I have a great cardiologist who knows nothing about diabetes, and I have a great dermatologist who knows nothing about diabetes, and I have an endocrinologist who knows nothing about the other things. So, for me, I'm always struggling with finding a physician who has some sensitivity to the interaction of diabetes with all the other potential problems that I have.

P2: In reference to what [P1] is saying, it's probably true in most cases that we have a lot of specialists . . . and, hopefully, what they're supposed to do is communicate with each other so that in the end they give you the best treatment.

### Time limitations and flexible participant involvement supports

Participants identified limited time as a barrier to diabetes management. Most frequently, participants attributed time limitations to balancing family and work responsibilities and with having limited time to exercise. One participant shared,

The biggest problem I have is time. I was used to eating on the run. Dinner often was through the drive-through. I had 2 children, I've got a demanding job, and I just kinda ran nonstop . . . and making a balance between taking care of myself and taking care of my family, handling responsibilities at work, is just constantly tugging at me.

Respondents suggested flexible participant involvement supports such as child care services, longer clinic hours, and programs that accommodate work schedules, which would facilitate participation in existing health programs. Additionally, participants discussed a need for pre-prepared diabetic meals and affordable, healthful convenience foods.

### Monetary barriers

Costs of medications and supplies were challenging for some participants. These issues were compounded in the case of participants who had no insurance, were underinsured, or required supplies that their insurance would not cover. However, the most frequently discussed monetary barrier related to the expense of eating healthful foods to manage diabetes. Participants discussed the need for monetary supports for medications, supplies, and healthful foods. The following interaction highlights this issue:

P1: It's more of the monetary support that I really need. Because of the way that I've changed my eating habits, I've noticed that a lot of the healthy food that I need to get to support my eating habits [is] expensive . . . and when I do buy this for myself, it's hard because I have 4 kids and a husband.

P2: For me, it would be the same problem. It's easier to get something for a dollar . . . at McDonald's. Fruit and vegetables are expensive.

## Discussion

This study provides insight into how employed adults perceive diabetes and the challenges to successful disease management. Participants in this study indicated that diabetes had pervasive emotional and physical effects on their lives. Additionally, physical and psychological barriers, time and monetary limitations, and a lack of social support complicated disease management. Participants in both the treatment group and the control group discussed the same barriers and service needs, even though participants in the treatment group had access to educational, motivational, dietary, and exercise supports.

Our results support previous recommendations to address social prejudice toward people with diabetes and to prevent potentially disabling complications through public awareness and education (12). Documented workplace discrimination allegations indicate that people with diabetes are more likely to experience prejudice, which can affect job retention (13). This in turn may affect access to health insurance and health maintenance. With regard to diabetes complications, a review that compared the benefits of science, surgery, service delivery, and social policy concluded that only public policies and workplace health initiatives focused on prevention can achieve the broad-scale changes needed to address diabetes (14).

Approximately 40% of America's national diabetes health care costs are expended on inpatient care for diabetes complications, although controlling blood glucose, blood lipids, and blood pressure greatly reduces the risk of developing these complications (1). Participants in this study rarely mentioned taking steps to avoid diabetes complications, implying a needed emphasis on active methods for prevention. People, especially those with little education, may not understand the progressive nature of diabetes (15). However, using diabetes complications as a scare tactic may only exacerbate feelings of helplessness if patients view future complications as inevitable.

Diabetes has pervasive effects on a person's life. However, our findings indicate that when people with diabetes interpret these effects positively, these feelings should be nurtured. Therapeutic approaches to enhance resiliency can supplement standard diabetes education (16), resulting in positive coping strategies, improved attitudes about living with the disease, and improved diet and exercise habits (17).

Although our participants did not frequently discuss reduced daily functioning, they did identify other emotional and health-related barriers, which could lead to future impairment. These findings support initiatives that incorporate social, emotional, and psychological supports into existing programs. The American Diabetes Association's *Standards of Medical Care* recommends that physician-coordinated teams include mental health professionals with interest and expertise in diabetes (18). Substantiated by previous research (19), psychological therapies improve long-term blood glucose control and alleviate psychological distress. Although psychological barriers to diabetes management are widespread, few patients report ever receiving psychological care. Furthermore, health care providers affirm that they do not have the resources to manage these problems (20). To be effective, programs should facilitate communication between all specialists involved in a patient's treatment and integrate psychological treatment into routine care to include diabetes support groups and one-on-one service.

Our results support previous findings that balancing familial and work responsibilities may complicate diabetes management because of feelings of obligation (6). Participants in this study needed flexible supports that facilitated program participation such as longer clinic hours, child care services, time management training, and flexible work schedules that accommodate doctor visits and exercise. Our results also reinforce a need for monetary support. Participants in our study did not offer concrete solutions to address the need for monetary support. However, on the basis of their conversations, proper disease management is costly and may be a factor when considering program development. Although new initiatives promote paying service providers to improve diabetes management, this does not support costs associated with maintaining individual lifestyle change and may exacerbate disparities in access to health care for less healthy patients and ethnic minorities (21).

Health care professionals and employers should continue to support people in effectively managing chronic illness to avoid serious repercussions (22). Our findings emphasize a need for greater public awareness and education, coordinated services that address emotional and other health-related barriers, and flexible supports that help people incorporate diabetes management into their lives. Additionally, the health care community should consider ways to support people with diabetes in maintaining positive lifestyle changes, which may be more cost-effective than simply implementing drug therapies (5).

These findings are generalizable to employed people with diabetes who represent a range of ethnic groups, including Asians and Pacific Islanders on Oahu. A limitation to our study is that our participants were volunteers and they had access to diabetes supports through the HI-DMIE; therefore, they may have been more motivated to manage their diabetes.

Our results indicate that diabetes supports should address the whole person — physically, psychologically, and socially. Future interventions for working people with diabetes should include coordinated programs that involve social, emotional, and lifestyle supports to help keep people healthy so that they can work well.

## Figures and Tables

**Table 1:** Characteristics of Focus Group Participants (N = 74), Hawaii, 2008

**Characteristic**	No. of Participants (%)
**Age, y**	
18-34	8 (11)
35-44	16 (22)
45-54	24 (32)
55-62	26 (35)
**Sex**	
Female	50 (68)
Male	24 (32)
**Diabetes type**	
Type 1	8 (11)
Type 2	65 (88)
Prediabetes	1 (1)
**HbA1c, %^a^**	
<7	26 (37)
7-9	30 (42)
>9	15 (21)
**Race/ethnicity**	
NHPI	28 (38)
Asian	24 (32)
White	10 (14)
Black	1 (1)
Mixed (non–NHPI)	9 (12)
Other	2 (3)
**Weekly hours worked^b^**	
≥40	23 (33)
20-39	42 (61)
1-19	3 (4)
Not working	1 (2)

Abbreviations: HbA1c, hemoglobin A1c; NHPI, Native Hawaiian/other Pacific Islander (part or full).

a N = 71.

b N = 69.

**Table 2:** Comparison of Focus Group Participants and Nonparticipants, by Age, HbA1c, and Diabetes Status, Hawaii, 2008

**Characteristic**	Participation Status	N	Value	*P* Value
Age, y, mean (SD)	Participants	74	48.7 (9.8)	.76^a^
	Nonparticipants	116	48.3 (9.7)	
HbA1c, mean % (SD)	Participants	71	7.7 (1.7)	.68^b^
	Nonparticipants	91	7.6 (1.6)	
Type 2 diabetes, n (%)	Participants	74	65 (88)	.52^c^
	Nonparticipants	116	98 (85)	

Abbreviations: SD, standard deviation; HbA1c, hemoglobin A1c.

a Calculated by using 2-tailed *t *test (*t_188_ =* 0.31).

b Calculated by using 2-tailed *t *test (*t_160_ =* 0.41).

c Calculated by using *χ^2^* test (*X* ^2^_*1*_ = 0.42).
